# Supplementary material for: The Southern Bluefin Tuna Mucosal Microbiome Is Influenced by Husbandry Method, Net Pen Location, and Anti-parasite Treatment
Source: Front Microbiol. 2020 Aug 24;11:2015. doi: 10.3389/fmicb.2020.02015 (PMC7476325; doi:10.3389/fmicb.2020.02015)
Supplement: Supplementary file 1 [file Data_Sheet_1.PDF]

frontiers

Supplementary Material

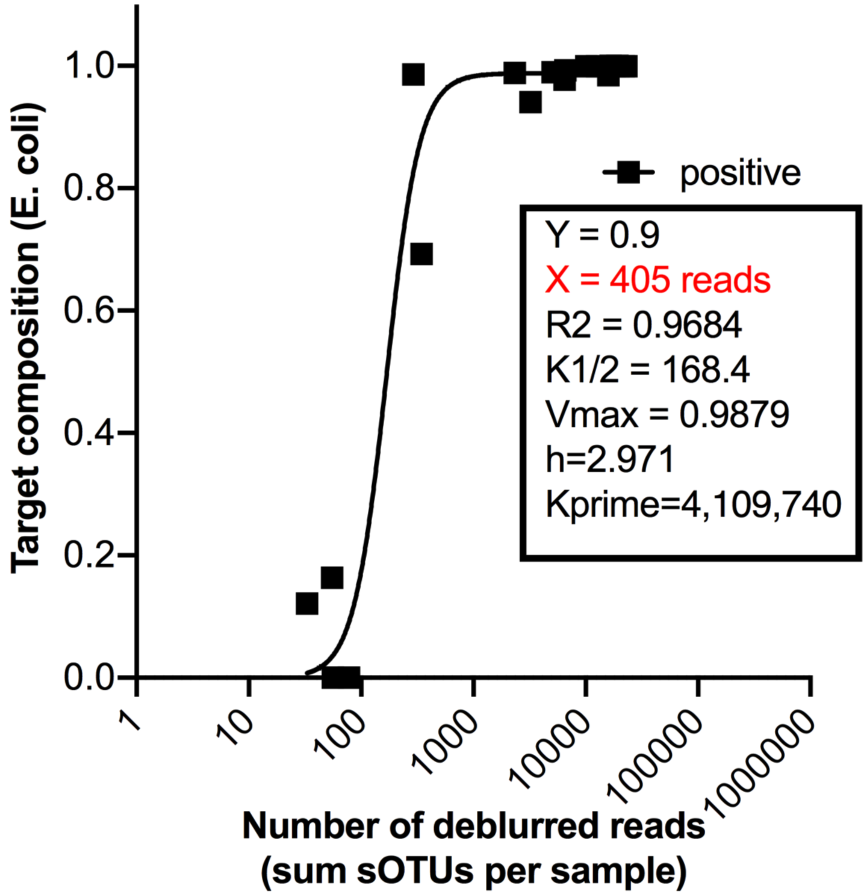

Supplementary Figure 1. Sample exclusion cut off determined by comparing relative abundaces of expected target sequences across a serial dilution of positive controls. See Katharoseq method for more details.

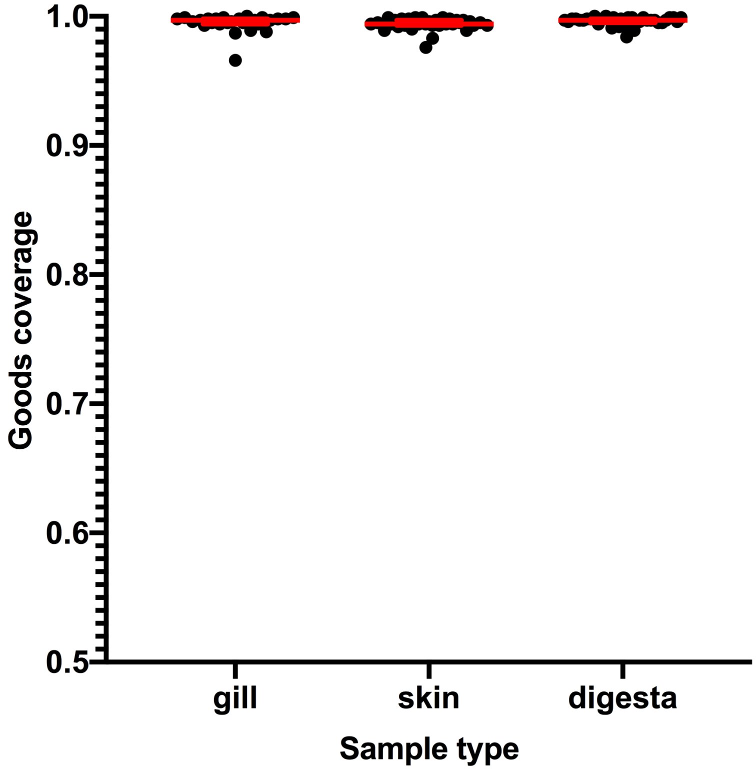

Supplementary Figure 2. Good's coverage (median, IQR) grouped by body site.

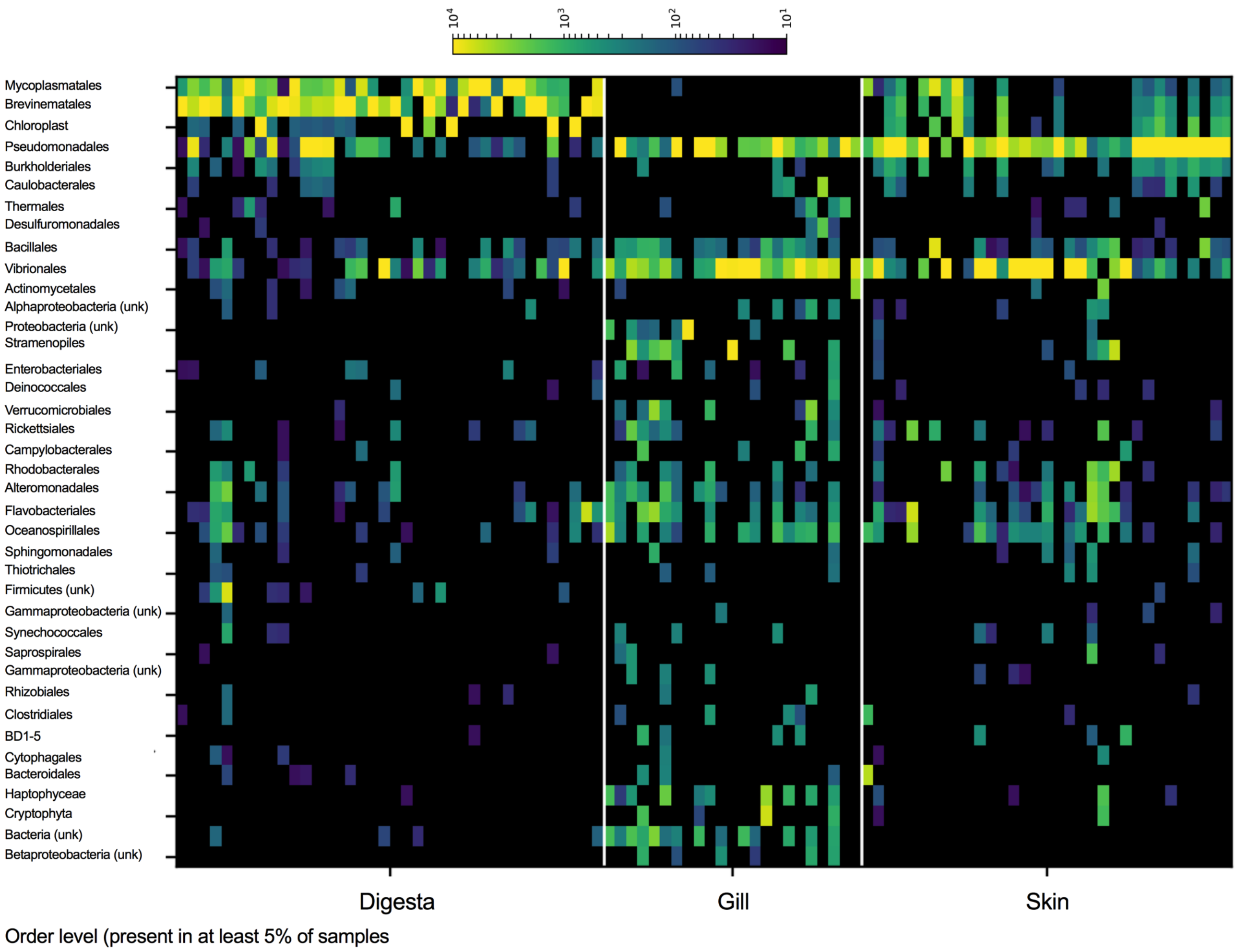

Supplementary Figure 3. Heatmap depiction of sOTUs collapsed or grouped at the order level. Orders which are present in at least 5% of the samples are included.

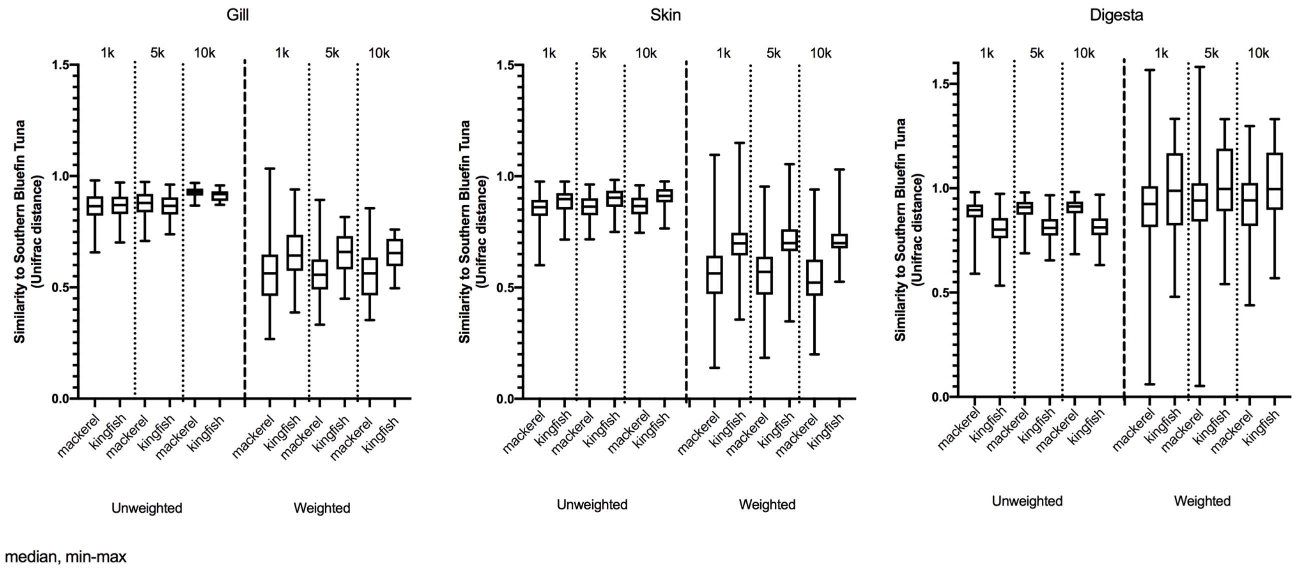

Supplementary Figure 4. Justification for setting a rarefaction depth of 1000 reads for cross-species comparisons of microbiome. Samples of gill, skin, and digesta are uniquely compared between fish species: mackerel and yellowtail kingfish to Southern Bluefin Tuna for Unweighted and Weighted Unifrac distances.
