# Supplementary figures and images for: The Southern Bluefin Tuna Mucosal Microbiome Is Influenced by Husbandry Method, Net Pen Location, and Anti-parasite Treatment
Source: Front Microbiol. 2020 Aug 24;11:2015. doi: 10.3389/fmicb.2020.02015 (PMC7476325; doi:10.3389/fmicb.2020.02015)

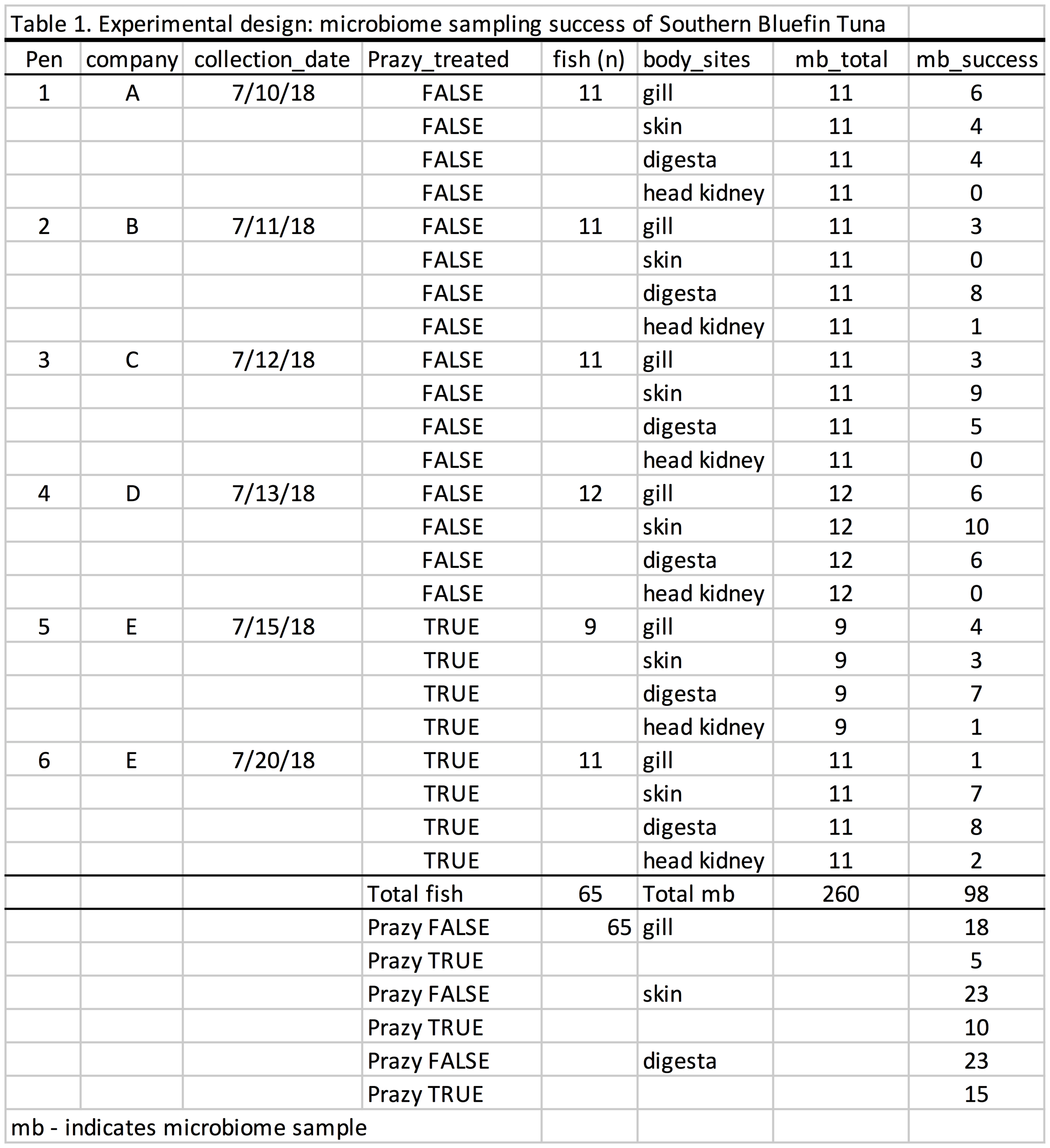

Supplement: Supplementary file 2 [file Image_1.TIFF]

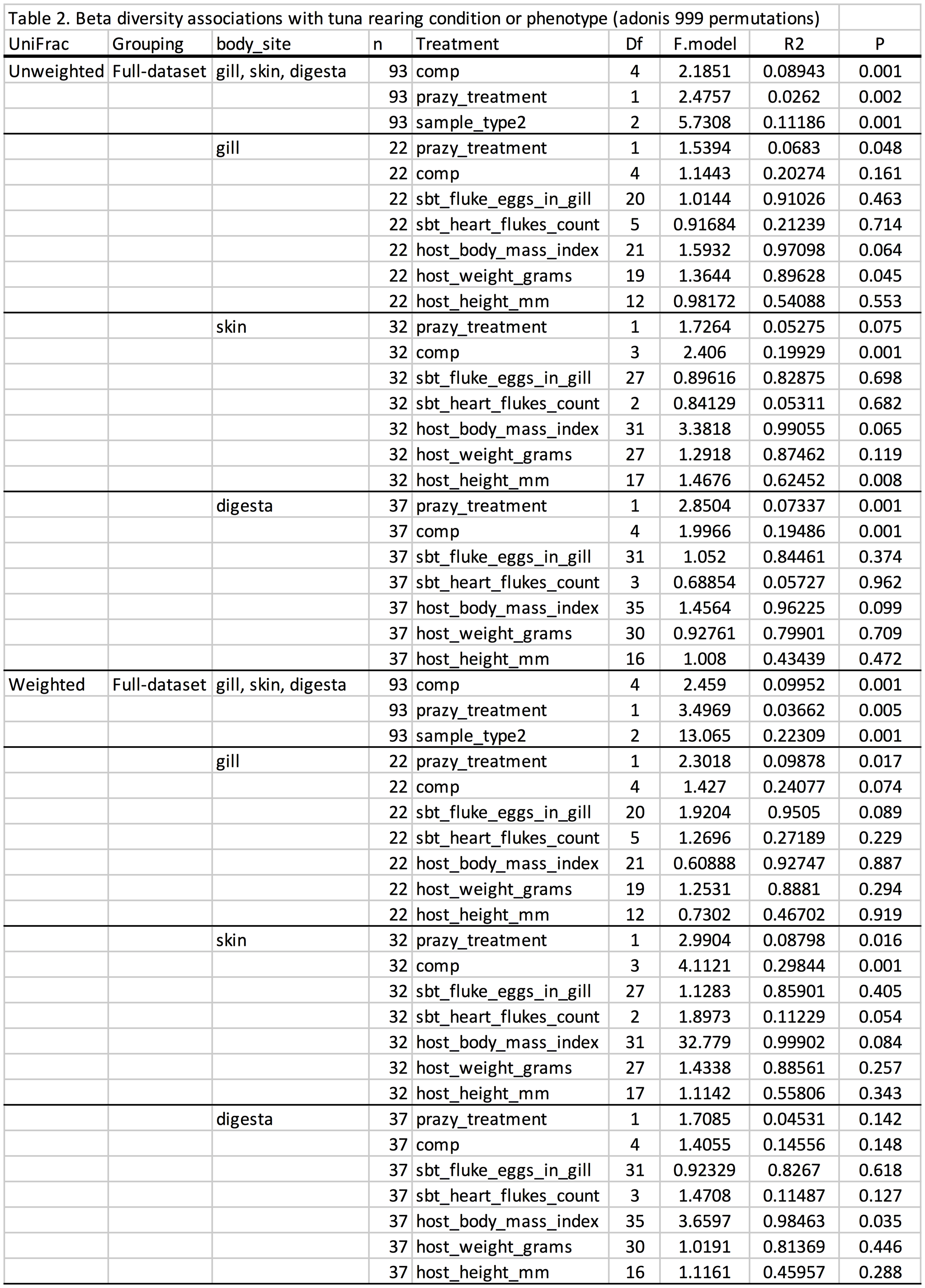

Supplement: Supplementary file 3 [file Image_2.TIFF]
